# Enhanced Electrical Transport Properties of Molybdenum Disulfide Field-Effect Transistors by Using Alkali Metal Fluorides as Dielectric Capping Layers

**DOI:** 10.1021/acsnano.3c11025

**Published:** 2024-04-08

**Authors:** Sumayah-Shakil Wani, Chen Chieh Hsu, Yao-Zen Kuo, Kimbulapitiya Mudiyanselage Madhusanka Darshana Kumara Kimbulapitiya, Chia-Chen Chung, Ruei-Hong Cyu, Chieh-Ting Chen, Ming-Jin Liu, Mayur Chaudhary, Po-Wen Chiu, Yuan-Liang Zhong, Yu-Lun Chueh

**Affiliations:** † Department of Materials Science and Engineering, National Tsing-Hua University, Hsinchu, 30013, Taiwan; ‡ Department of Physics and Quantum Information Center, Chung Yuan Christian University, Taoyuan, 32034, Taiwan; § College of Semiconductor Research, National Tsing-Hua University, Hsinchu, 30013, Taiwan; ∥ Institute of Electronics Engineering, National Tsing Hua University, Hsinchu, 30013, Taiwan; ⊥ Department of Physics, National Sun Yat-Sen University, Kaohsiung, 80424, Taiwan; # Department of Materials Science and Engineering, Korea University, Seoul 02841, Republic of Korea

**Keywords:** molybdenum disulfide, monolayer, field-effect transistor, alkali fluoride capping, doping, 2D semiconductors

## Abstract

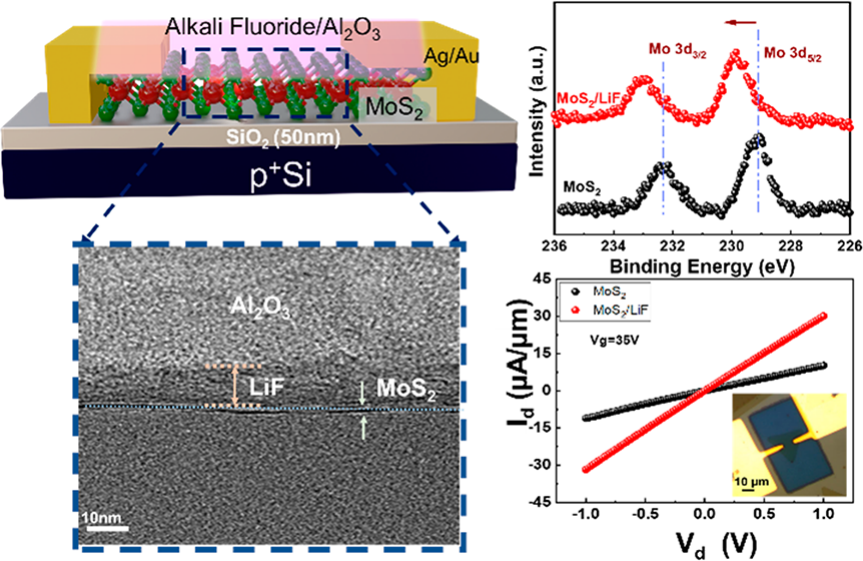

The electronic properties of 2D materials are highly influenced by the molecular activity at their interfaces. A method was proposed to address this issue by employing passivation techniques using monolayer MoS_2_ field-effect transistors (FETs) while preserving high performance. Herein, we have used alkali metal fluorides as dielectric capping layers, including lithium fluoride (LiF), sodium fluoride (NaF), and potassium fluoride (KF) dielectric capping layers, to mitigate the environmental impact of oxygen and water exposure. Among them, the LiF dielectric capping layer significantly improved the transistor performance, specifically in terms of enhanced field effect mobility from 74 to 137 cm^2^/V·s, increased current density from 17 μA/μm to 32.13 μA/μm at a drain voltage of *V*_d_ of 1 V, and decreased subthreshold swing to 0.8 V/dec The results have been analytically verified by X-ray photoelectron spectroscopy (XPS) and Raman, and photoluminescence (PL) spectroscopy, and the demonstrated technique can be extended to other transition metal dichalcogenide (TMD)-based FETs, which can become a prospect for cutting-edge electronic applications. These findings highlight certain important trade-offs and provide insight into the significance of interface control and passivation material choice on the electrical stability, performance, and enhancement of the MoS_2_ FET.

## Introduction

As a group of semiconductors with atomically thin structures and great mobility, two-dimensional (2D) transition metal dichalcogenides (TMDs) have garnered a great deal of interest. They are being considered as potential substitutes for graphene in various applications.^1−3^ TMDs have a layered structure consisting of monolayers, similar to stacked graphene sheets, but with interactions between layers through interplanar van der Waals forces.^4^ One advantage of TMDs over graphene is that they possess a semiconductor-like energy gap.^5^ This bandgap means that TMDs can control the flow of electrons more effectively, making them suitable for practical 2D-based devices. Molybdenum disulfide (MoS_2_), among the TMDs, stands out as one of the most meticulously explored materials, owing to its abundant availability and widespread research interest. Its substantial bandgap and extraordinary characteristics make it a top contender as a channel material in future energy-efficient field-effect transistors (FETs).^6−9^ Atomic thickness and the ideally dangling bond-free surface of MoS_2_ make it promising for van der Waals assembly on various materials or substrates. Furthermore, this property contributes to mitigating short-channel effects, making MoS_2_ a viable option for use as a semiconducting channel material in nanoscale electronics and optoelectronic devices.^3,10−12^ When scaled down, MoS_2_ FETs have demonstrated an impressive on/off ratio, high carrier mobility, and low power consumption.^13−19^ Extensive experimental study aimed at improving MoS_2_ FET performance has been motivated by these properties.

To implement MoS_2_ monolayers in practical electronic devices, it is crucial to tailor the device properties to achieve enhanced output characteristics. Therefore, the design of the device structure should prioritize stability. Many MoS_2_ FETs studied so far have utilized a primary back-gated architecture, for which the surface of the MoS_2_ channel is exposed and vulnerable to the adsorption of water molecules and oxygen, leading to an increased hysteresis in devices because of undesirable effects such as interface trap density.^20−26^ Qiu et al. reported that back-gated bilayer MoS_2_ FETs are susceptible to ambient oxygen and water molecules/moisture exposure, affecting their conductivity and field-effect mobility.^27^ Therefore, these transient effects must be mitigated, and effective strategies must be identified to ensure device stability. However, various methods have been explored to overcome these issues, including modifying the substrate chemistry beneath the channel,^28−30^ fully encapsulating the channel with a high-*k* dielectric (such as atomic layer deposition (ALD) grown aluminum oxide or hafnium oxide), using a 2D insulator like hexagonal boron nitride,^31−34^ and repairing sulfur vacancy defects in the MoS_2_ lattice.^35−42^ However, finding industry-compatible methods remains a significant challenge.^43−45^

An effective way of reducing ambient exposure is to encapsulate the MoS_2_ FET channel with extra protection layers. Despite numerous studies on various capping layers for MoS_2_ FETs, their efficacy in preventing molecular adsorption has received limited attention, with most studies focusing on enhancing field-effect mobility through the modification of the MoS_2_–dielectric interface. Surface passivation using capping layers, such as Al_2_O_3_,^46^ HfO_2_,^47^ or hexagonal boron nitride (hBN),^34^ offers an effective solution for addressing challenges in 2D materials. Encapsulation ideas with these materials aim to isolate MoS_2_ from the air and enhance the FET device performance. Due to the lack of surface dangling bonds on MoS_2_, atomic-layer-deposited oxide capping layers exhibit nonuniform growth and struggle to achieve full coverage, especially with ultrathin protective layers, despite the extended deposition process.^48^ Encapsulation with exfoliated hBN involves intricate manipulations and is impractical for scalable processing.^49^ Another study explored C_60_ and MoO_3_ as surface modification layers, with C_60_ having negligible influence on MoS_2_ FET devices, while MoO_3_ induces significant charge transfer, depleting electron charge carriers in MoS_2_ FET devices.^50^ Although the C_60_ layer shows some promise as a surface protection layer, no study has investigated its ability to reduce the effects of ambient atmosphere effects. Alternatively, organic molecules, especially phthalocyanine and its metal derivatives, were assembled on TMD surfaces through solution-process and vapor-phase methods for the passivation of surface defects. However, achieving atomic uniformity on MoS_2_ with these protective molecules remains challenging, and thickness control remains uncontrollable, hindering their protective ability.^51^ Another study explored SiNx as a passivation layer deposited through plasma-enhanced chemical vapor deposition (PECVD), potentially damaging MoS_2_ during deposition.^52^ Some researchers proposed that poly(methyl methacrylate) (PMMA) films can form air pockets on 2D material surfaces, leading to oxidation and degradation over extended periods.^51^ Another group investigated the n-type doping of MoS_2_ FETs using a poly(vinyl alcohol) (PVA) coating, a water-soluble polymer.^53^ As expected, PVA tends to trap water, degrading the effectiveness and stability of the doping mechanism, emphasizing the need for careful consideration in choosing passivation strategies. Different fluorinated copolymers such as CYTOP^54^ and p(V4D4-coCHMA)^55^ have been studied as passivation layers to shield the MoS_2_ FET channel from the environment.

In this study, a precisely controlled approach was introduced for selectively n-doping monolayer (1L) MoS_2_ through the implementation of a lithium fluoride (LiF) capping layer. Although LiF capping layers are not commonly used in 2D electronics, researchers anticipate positive outcomes when applied to MoS_2_ FETs. The incorporation of LiF in MoS_2_ FETs is expected to enhance electron injection into the MoS_2_ channel, thereby improving the overall electrical device characteristics. Our work not only concentrates on enhancing device performance but also contributes to the successful passivation of FETs, thereby reducing hysteresis. By adjusting the thickness of the LiF capping layer, there is potential to optimize electron injection and enhance the efficiency of MoS_2_ FETs. Validation through analytical techniques, such as X-ray photoelectron spectroscopy (XPS), Raman spectroscopy, and photoluminescence (PL) spectroscopy, confirmed the results. LiF capping layers of varying thicknesses on back-gated MoS_2_ transistors showed n-type doping behavior, as evidenced by shifts in threshold voltages (*V*_th_) and a significant increase in current density from 17 to 32.13 μA/μm at a drain voltage (*V*_d_) of 1 V. To explore the intrinsic electronic transport properties of TMD FETs, the study examined the effects of gate and drain bias on 1L MoS_2_ FETs encapsulated with different capping layers, including lithium, sodium, and potassium alkali fluorides.

## Results and Discussion

**Figure 1:**
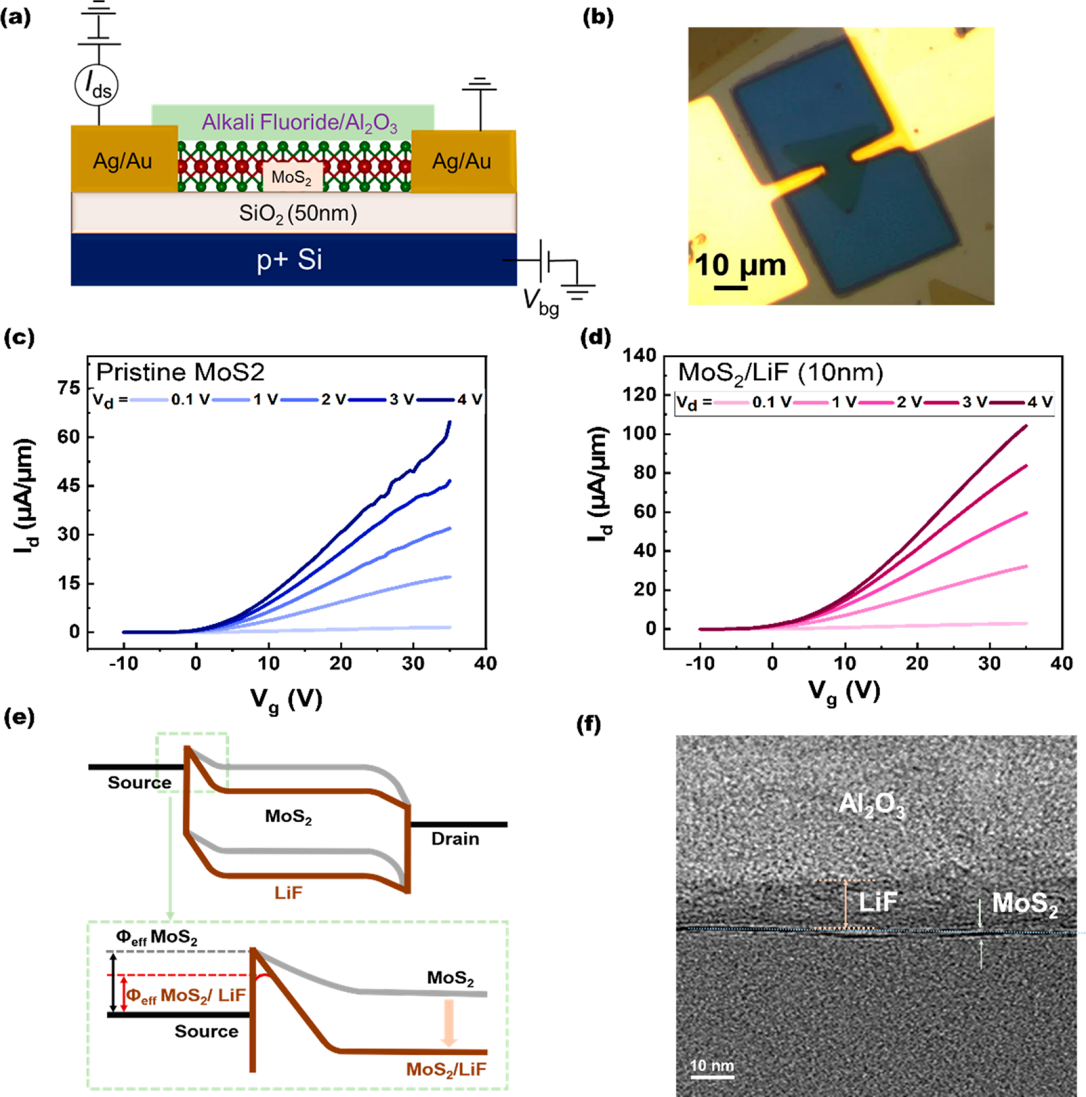
(a) Schematic description of a LiF capping layer on MoS_2_ FETs. (b) An OM image of the LiF-capped MoS_2_ FET. *I*_d_–*V*_g_ curves of the MoS_2_ FET of (c) without and (d) with the LiF capping layers. (e) Energy band diagram of the MoS_2_ FET channel before and after the capping of the LiF layer. (f) A cross-sectional TEM image of the LiF-capped MoS_2_ FET. Al_2_O_3_ only acts as the protective layer to prevent the etching away of the LiF capping layer after the lift-off process.

Figure 1a shows a cross-sectional schematic of fabricated transistors by transferring a chemical vapor deposition (CVD)-synthesized 1L MoS_2_ on a 50-nm-thick SiO_2_/Si substrate (Figures S1 and S2) (details can be found in the Methods section). The Si substrate is highly doped (p++) silicon, functioning as a back gate, while Ag/Au served as a contact material. Alkali fluoride acts as a main doping layer in contact with the MoS_2_ channel. The top layer of Al_2_O_3_ was used to protect the alkali fluoride layer during the lift-off process and will be discussed in detail later. Figure 1b shows an optical microscopy (OM) image of a fabricated 1L MoS_2_ FET (*L* = 10 μm, *W* = 3 μm) with capping layers. The shape of an electrode has a dual impact on the mobility of charge carriers and the distribution of the electric field within the material. The surface area and contact resistance of the electrode shape directly influence charge carrier mobility. The larger surface area provides more contact points for efficient charge transfer, enhancing mobility. Moreover, optimization of electrode shape reduces contact resistance, enabling better electrical contact and promoting higher mobility. Careful design enhances the charge carrier transport with a uniform field distribution, while nonuniform fields cause variations in carrier properties. Note that the edge effect impacts carrier mobility, but smoothing the edge mitigates this. As a result, electrode shape is critical for carrier transport and electrical properties. Therefore, in our fabrication process, we preferred the simple electrode shape to achieve the desired carrier transport characteristics in electronic devices, in conjunction with considering other factors such as material properties and device design.^56^Figure 1c and d represent the *I*_d_–*V*_g_ performance of the monolayer MoS_2_ FET before and after the LiF capping layer, respectively. Capping the channel of the FET shows significant improvement in its electrical performance, for which enhanced on-current and mobility of 32.13 μA/μm and 137 cm^2^/(V s), respectively, can be achieved. Figure 1e shows the schematic of the energy band diagram of MoS_2_ and MoS_2_/LiF, representing the band bending before and after n-type doping in the 1L MoS_2_ channel layer. After applying doping to the MoS_2_ device, the energy band of the MoS_2_ shifts downward because the n-type doping transfers electrons to MoS_2_, which will be proved and supported in the upcoming data analysis. The thickness of the LiF capping layer (best-optimized device) between the MoS_2_ channel and the Al_2_O_3_ protective layer was identified through high-resolution transmission electron microscopy (HRTEM), as shown in Figure 1f. The interlayer spacing of MoS_2_, approximately 0.7 nm, was revealed through the preparation of a cross-sectional transmission electron microscopy (TEM) sample by using a focused ion beam (FIB) technique. Additionally, it was observed that a 10-nm-thick LiF capping layer fully encapsulates the monolayer MoS_2_.

**Figure 2:**
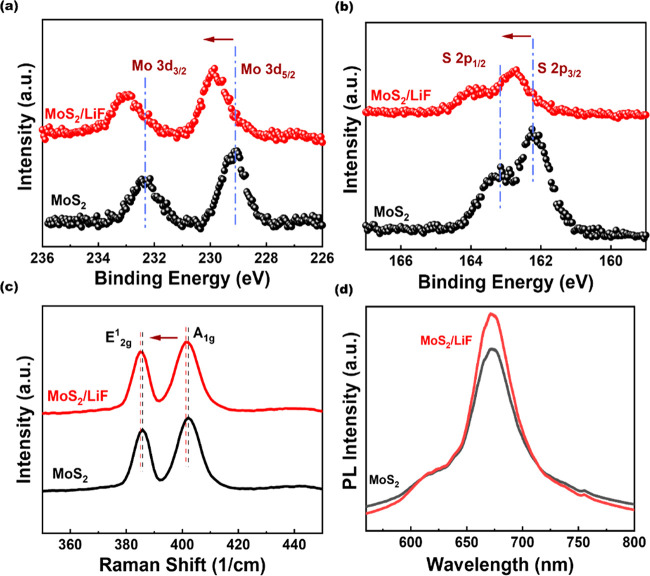
X-ray photoelectron spectroscopy (XPS) spectra of (a) Mo 3d and (b) S 2p before and after the capping of the LiF layer. The shift of the binding energies to higher energy indicates n-type doping. (c) Raman spectra of MoS_2_ before and after the capping of the LiF layer. The LiF deposition induces a red-shift behavior on (d) photoluminescence (PL) measurements of MoS_2_ before and after the capping of the LiF layer.

To investigate the valence state of MoS_2_ before and after the LiF capping layer, XPS surface analysis was conducted. Figure 2a and b show Mo 3d and S 2p peaks before and after the LiF capping layer. Following the LiF capping layer, noticeable shifts toward a higher binding energy were observed in Mo 3d and S 2p peaks. Specifically, the Mo 3d_3/2_ peak shifts from 232.0 to 233.0 eV, the Mo 3d_5/2_ peak shifts from 229 to 229.8 eV, the S 2p_1/2_ peak shifts from 163.15 to 163.8 eV, and the S 2p_3/2_ peak shifts from 162.25 to 162.7 eV. These upward shifts of the peaks are directly attributed to the n-doping, resulting in a shift of the Fermi level toward the conduction band edge. The blue shift observed in the binding energy of the elemental electrons is considered to be a direct indication of surface charge transfer doping. A similar energy shift toward higher binding energy values in the XPS spectra was observed in the MoS_2_ layer capped with the NaF layer, as shown in Figure S3a and b, confirming the occurrence of the n-type doping. Furthermore, Raman and PL spectroscopy were employed to investigate the doping effect in the 1L MoS_2_ layer, as shown in Figure 2c and d. The red shift in typical characteristic vibrational modes, i.e., E^1^_2g_ and A_1g,_ peaks, can be clearly observed, confirming the n-type doping after the LiF capping. The same trend can be observed for the case of the NaF-capped MoS_2_ FET, as shown in Figure S3c and d.

**Figure 3:**
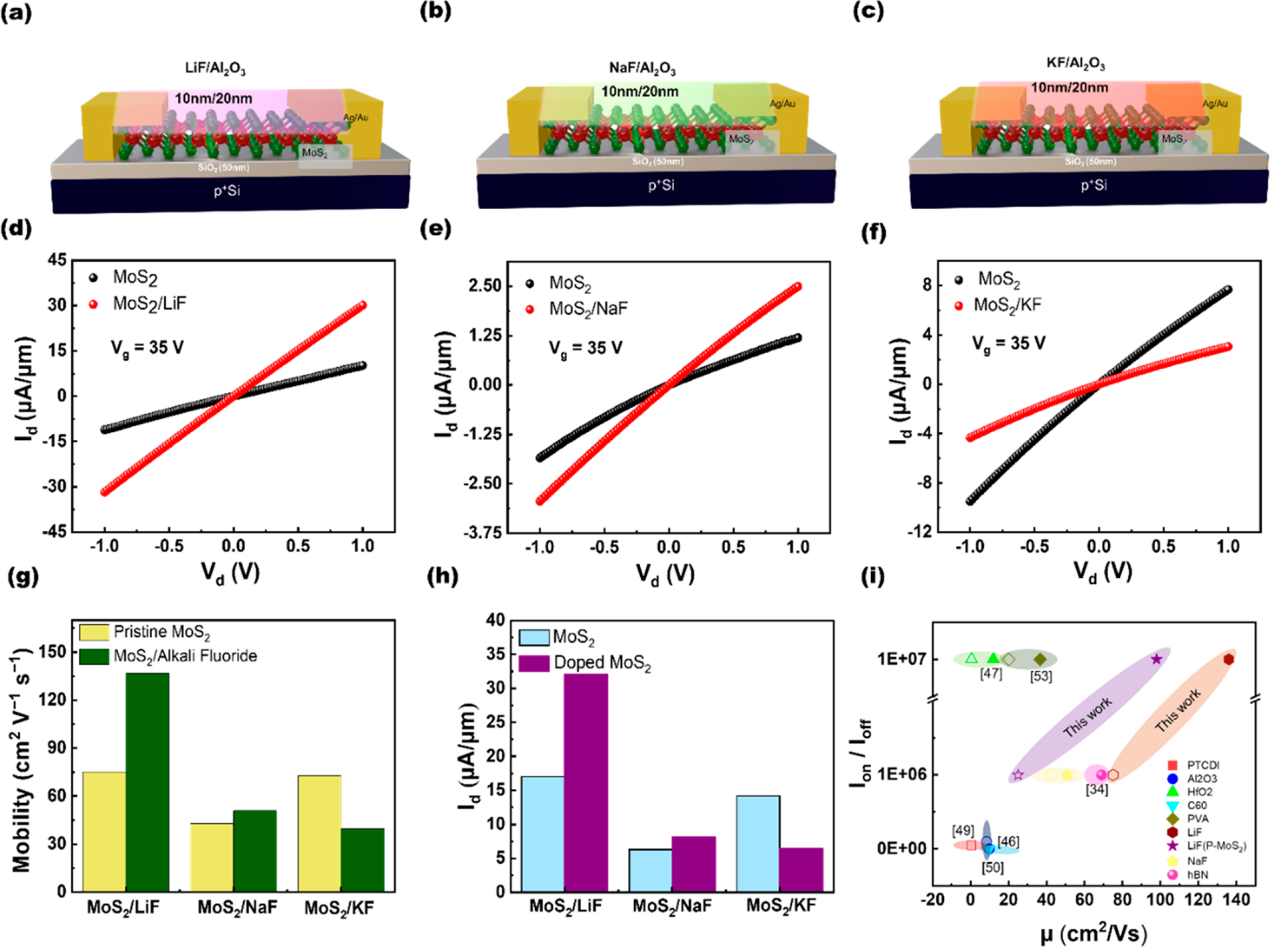
Schematic diagram of the (a) 10-nm-thick LiF, (b) 10-nm-thick NaF, and (c) 10-nm-thick KF layers on MoS_2_ FETs. Output characteristics of (d) 10-nm-thick LiF, (e) 10-nm-thick NaF, and (f) 10-nm-thick KF layers on MoS_2_ FETs. Performance comparison for different alkali metal layers on MoS_2_ FETs. (g) The trend in mobility, (h) charge density, and (i) maximum μ (cm^2^/(V s)) vs *I*_on_/*I*_off_ of our LiF-doped MoS_2_ compared with other doped 2D-FETs. The empty and solid symbols represent the pristine and doped MoS_2_.

To investigate the effect of alkali fluoride doping on the MoS_2_ FET, we fabricated several devices with different capping layers, and electrical measurements were obtained before and after the capping layers. Figure 3a to c demonstrate schematics of the MoS_2_ FETs with different capping layers, including lithium fluoride (LiF), sodium fluoride (NaF), and potassium fluoride (KF) layers, respectively. Thicknesses for all three capping layers were kept constant, i.e., 10 nm. To protect this layer from etching away during the lift-off processes, the capping layer of 20-nm-thick Al_2_O_3_ film was deposited. Alkali metals (Li, Na, and K) react with fluorine (F) to form ionic compounds, and this process is influenced by factors such as electronegativity, electron affinity, and ionization energy. Electronegativity means the ability of an atom to attract electrons in a chemical bond. Fluorine has the highest electronegativity, while alkali metals have low values. The alkali metal donates an electron to fluorine in ionic compounds, resulting in the formation of a cation (Li^+^, Na^+^, K^+^) and an anion (F^–^) due to differences in electronegativity. Since fluorine has high electron affinity (tendency to gain electrons) and high ionization energy (energy required to remove an electron from an atom or ion), it prefers to gain electrons, forming an ion. In contrast, alkali metals tend to lose an electron and become cations because of their low electron affinity and ionization energy. The resulting ions (Li^+^, Na^+^, K^+^), and (F^–^) are attracted to each other due to electrostatic forces and form an ionic compound. Overall, the high electronegativity of fluorine, combined with the low electron affinity and ionization energy of alkali metals, promotes the formation of ionic compounds between alkali metals and fluorine. All these parameters are summarized in Supplementary Table 1.^57,58^Figure 3d to f show *I*_d_–*V*_d_ output characteristics of 1L MoS_2_ back-gated FETs with and without the capping of LiF, NaF, and KF layers at a *V*_g_ of 35 V. Note that the doping by the LiF capping layer offers better gate control over MoS_2_ FETs compared to MoS_2_ FETs with the doping by NaF and KF capping layers. LiF forms a more uniform and well-defined interface with MoS_2_, minimizing the formation of charge traps and interface roughness and thus enhancing its performance. After the doping by the capping of LiF and NaF layers, the devices show enhanced device performance. Consequently, the Schottky behavior diminishes, and the low field regime (*V*_d_ = −1 to 1 V) exhibits an ohmic behavior. As per the electrical performance, the 1L MoS_2_ back-gated FETs by the capping of the LiF layer proved the most favorable and the 1L MoS_2_ back-gated FETs by the capping of the NaF layer also had a positive impact (Figure S4). However, the 1L MoS_2_ back-gated FETs by the capping of the KF layer had a detrimental impact on the MoS_2_ devices (Figure S5). The KF doping, employed to modify the electronic characteristics of MoS_2_ FETs, can yield complex effects on electrical performance. We assume that the introduction of the KF capping layer can induce defects within the MoS_2_ lattice, acting as scattering centers that hinder charge carrier mobility and impede performance. Charge traps arising from these defects can capture and release charge carriers, extending trapping and detrapping times and ultimately reducing carrier mobility. Scattering mechanisms, including charged impurity scattering and phonon scattering, can also contribute to a decrease in carrier mobility. The effects of KF doping on MoS_2_ FETs are contingent upon nuanced experimental conditions and intended device objectives, emphasizing the need for thoughtful optimization and exploration to balance the potential benefits and drawbacks of doping strategies.

Figure 3g and h show the performance comparison between three capping layers. The current density at *V*_d_ = 1 V increases significantly from ∼17 to ∼32 μA/μm for the 1L MoS_2_ back-gated FETs by the capping of the LiF layer. The current density of 1L MoS_2_ back-gated FETs by the capping of the NaF exhibit a slight increase to ∼8 μA/μm from ∼6 μA/μm, while the current density of 1L MoS_2_ back-gated FETs by the capping of the KF layer decreases to ∼6.5 μA/μm from a pristine value of ∼14 μA/μm at *V*_d_ = 1 V. A similar trend was observed for the mobility and the on-current. This trend can be explained by considering the structural properties and compatibility of these capping layers with the MoS_2_ material, with which the LiF has a smaller ionic radius compared to that of NaF and KF. The smaller ionic radius of Li^+^ ions in LiF allows for better lattice matching with the MoS_2_ crystal structure.^59^ This facilitates a more seamless integration of the LiF into the MoS_2_ lattice, minimizing lattice strain and defects that can impede carrier mobility. A lower defect density reduces scattering and enhances carrier mobility, leading to improvement of conductivity and device efficiency. Since the LiF has the smallest ionic radius, it will exhibit a stronger ionic character than that of NaF and KF. This implies that the difference in electronegativity between lithium (Li) and fluorine (F) is larger than that between sodium (Na) or potassium (K) and fluorine, leading to a higher degree of charge transfer from LiF to the MoS_2_ material, resulting in the enhancement of the doping efficiency with higher density of charge carriers in the MoS_2_ channel, improving overall device performance. Based on these factors, the smaller ionic radius, enhanced charge transfer, and lower defect density associated with the capping of the LiF layer make it a superior choice compared to the capping of NaF and KF layers for better device performance.^60^ To confirm that the Al_2_O_3_ top layer does not play an important role in influencing the device performance, we fabricated devices with only Al_2_O_3_ as the passivation/capping layer, as shown in Figure S6. Although the device does show performance improvement, it is not as significant as that obtained by the capping of the LiF layer. Also, subthreshold swing (SS) increases after the capping of the Al_2_O_3_ film. In Figure 3i, we have compared our FET performance with the previous studies of passivated or doped MoS_2_ FET.^34,46,47,49,50,53^

**Figure 4:**
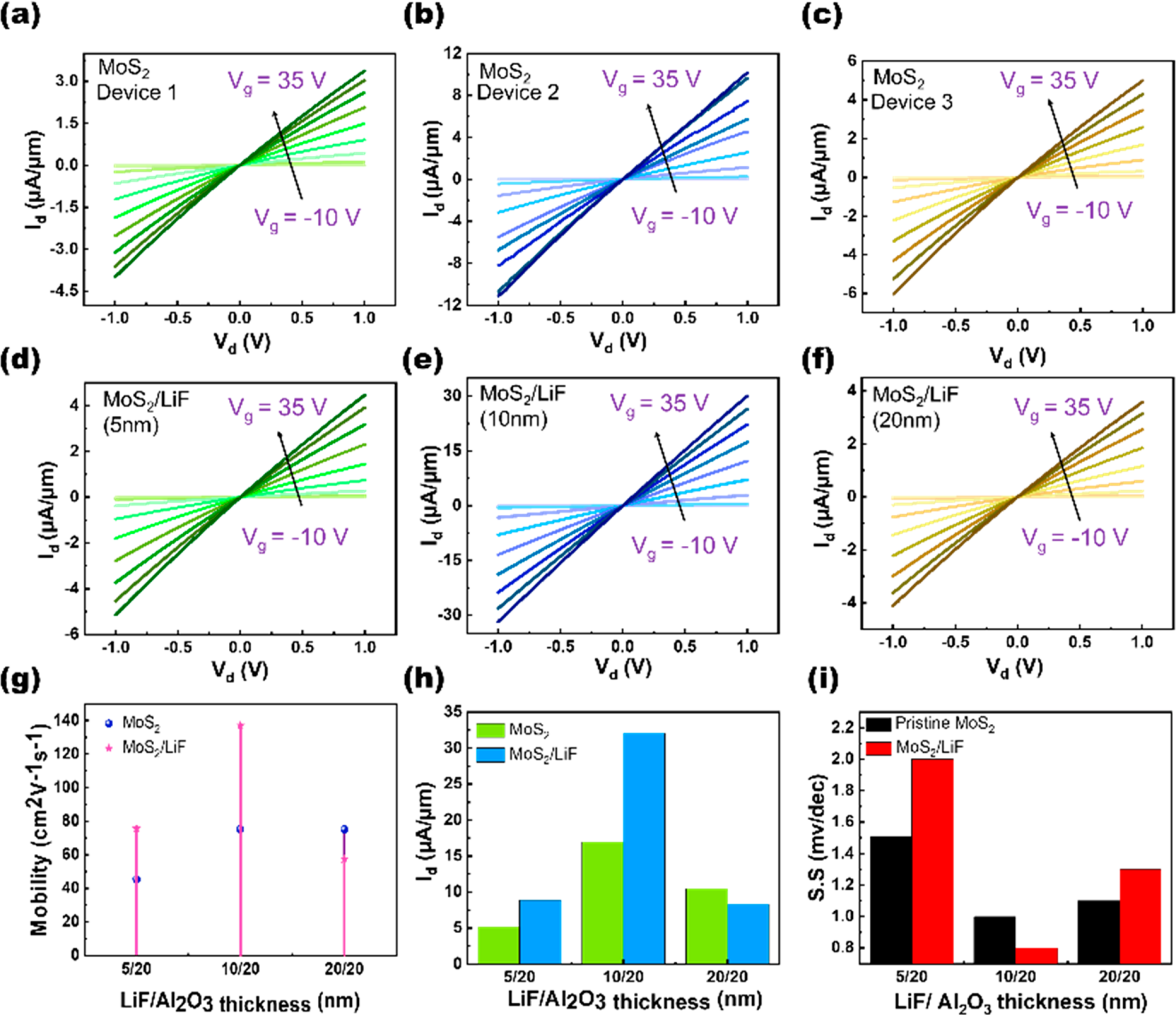
(a–c) Transfer characteristics of pristine MoS_2_ FET device 1, 2, and 3, respectively. Transfer characteristics for the MoS_2_ FET after LiF doping with different thicknesses: (d) 5-nm-thick LiF, (e) 10-nm-thick LiF, and (f) 20-nm-thick LiF layers. (g–i) Performance comparison for the different thicknesses of LiF layers on MoS_2_ FETs.

For a better understanding of the doping effect by the capping of the LiF layer, we fabricated devices with different thicknesses of LiF capping layers. Figure 4a to c display *I*_d_*–V*_d_ output characteristics of three pristine monolayer devices 1–3 measured at *V*_g_ = −10 to 35 V with an increment of 5 V at the low-field regime (*V*_d_ = −1 to 1 V). Figure 4d to f can be referred to as devices 1–3 with 5-, 10-, and 20-nm-thick LiF capping layers, respectively. Devices 1 and 2 with 5- and 10-nm-thick LiF capping layers revealed good linearity at the low-*V*_d_ regime, as shown in Figure 4d to e. However, when the thickness increases to a 20-nm-thick LiF capping layer, the device performance degrades, as shown in Figure 4f. The degree of the doping concentration significantly influences the impact, and excessive doping concentrations can generate numerous defects that severely disrupt the crystal structure, thus degrading electrical properties. The carrier mobility (μ) for all devices was extracted from *I*_d_–*V*_g_ (Figure S7a to c) slopes in the linear region and calculated using the following equation:
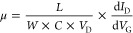
1where *L*, *W*, *C*, *V*_D_, *I*_D_, and *V*_G_ are the channel length, the contact width, the capacitance of the gate oxide, the drain voltage, the drain current, and the gate voltage, respectively. At *V*_d_ = 1 V, the mobility increases from 45.4 cm^2^/(V s) to 75.6 cm^2^/(V s) for the 5-nm-thick LiF capping layer. For a 10-nm-thick LiF capping layer, the enhanced mobility from 74 to 137 cm^2^/(V s) can be achieved. On the other hand, for 20-nm-thick LiF-capping devices, mobility decreases to 57.5 cm^2^/(V s) from the pristine value of 75.1 cm^2^/(V s) at *V*_d_ = 1 V, as shown in Figure 4g. Figure 4h and i display a similar trend in on-current (*I*_on_) and the subthreshold swing. The SS for the 10-nm-thick LiF-capped FET decreases to 0.84 V/dec from 0.9 V/dec but increases to 1.25 V/dec for the FET with the 20-nm-thick LiF capping layer. An on–off ratio of ∼10^7^ was obtained for the best-optimized device with a 10-nm-thick LiF capping layer. The on/off ratio can be calculated from the transfer curves in a logarithmic scale, as shown in Figure S8a–c. The enhancement in device performance after capping of the LiF layer can be anticipated due to the intercalation of lithium (Li^+^) ions from the LiF layer into the MoS_2_ lattice. During this intercalation process, the Li^+^ ions migrate into the MoS_2_ material and occupy interstitial sites, introducing additional charge carriers. The intercalated Li^+^ ions donate electrons to the MoS_2_ layer, increasing the electron concentration within the MoS_2_ channel. These additional electrons become mobile charge carriers, modulating the carrier concentration in the MoS_2_ FET device. Consequently, the electrical properties of the device are altered. Based on the electrical performance of our devices after the LiF capping of the channel, we observed n-type doping. This result can be proved by the negative threshold voltage (*V*_th_) shift obtained from the transconductance curve, as shown in Figure S8d to f. The *V*_th_ shift for the best-optimized device with a capping layer of 10-nm-thick LiF demonstrated a shift from −7.5 to −10 V at the *V*_d_ = 1 V.

**Figure 5:**
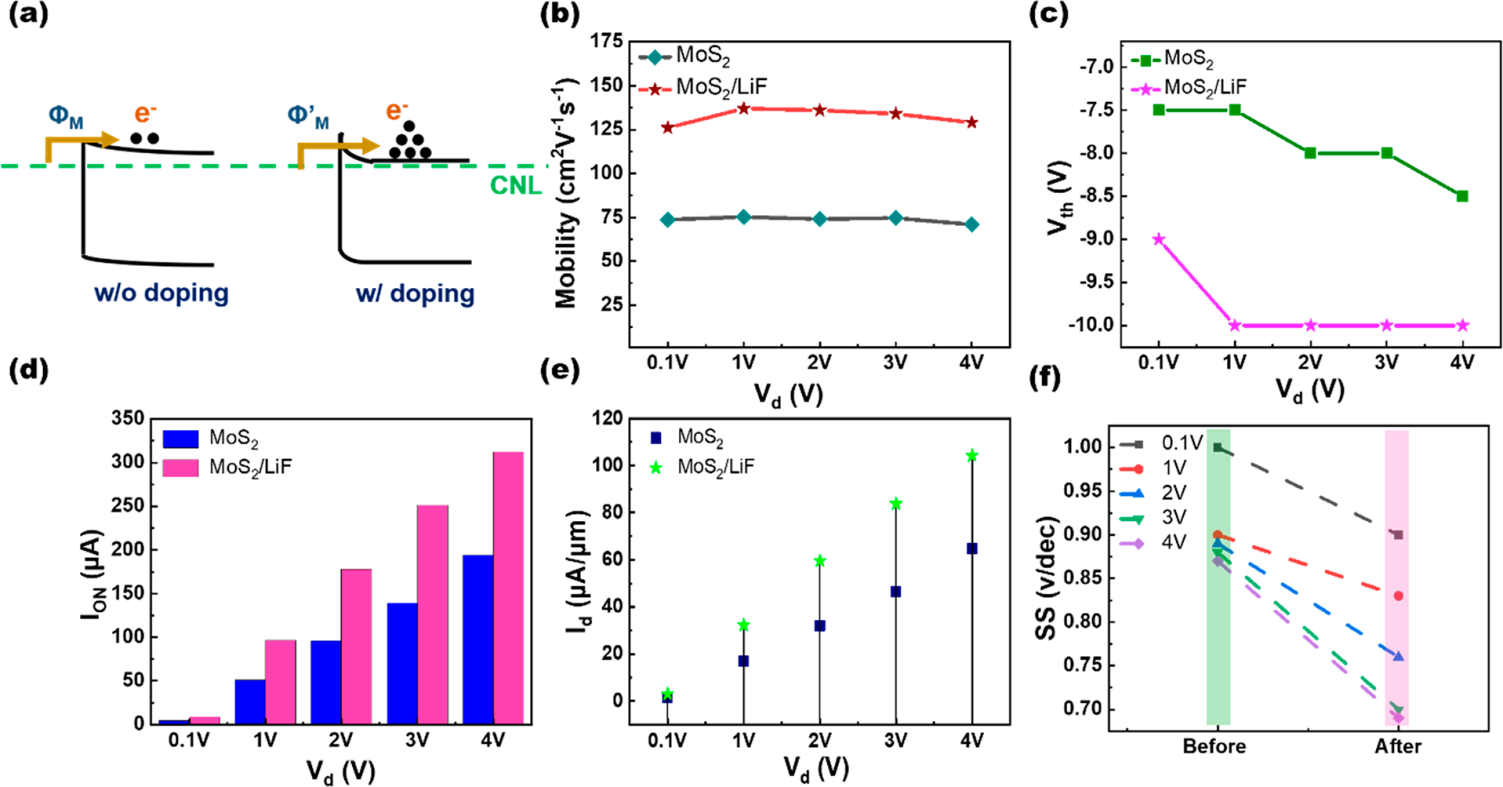
(a) Schematic energy band diagrams of the MoS_2_–LiF interface illustrating a proposed mechanism of electron doping. Electrical performance of a 10-nm-thick LiF layer on MoS_2_ FETs at different *V*_d_. (b) Mobility, (c) threshold voltage, (d) on-current, (e) charge carrier density, and (f) subthreshold swing before and after the capping of the LiF layer.

By adjusting the deposition parameters, specifically different thicknesses of LiF capping layers, it is possible to influence the efficiency of doping and subsequently control the carrier concentration within the MoS_2_ channel. There are several factors contributing to the decrease in performance, with an increase in the thickness of 20 nm. First, a thicker LiF layer introduces more scattering centers for charge carriers in the MoS_2_ channel, which hampers the mobility of charge carriers, resulting in reduced device performance. Furthermore, a thicker LiF capping layer tends to trap more charge carriers at the interface between LiF and MoS_2_, in which charges modify the electrostatics of the device, distorting the electric field and reducing the device performance. These defects can scatter charge carriers (electrons or holes) as they move through the channel of the FET. This scattering reduces the charge carrier mobility, which is a measure of how quickly charge carriers can move through the semiconductor channel. Lower mobility can lead to reduced current flow and, consequently, reduced device performance. This result can manifest as a reduced on/off current ratio and an increased subthreshold swing. Moreover, increasing the LiF layer thickness introduces interface effects such as charge transfer and dipole formation at the LiF/MoS_2_ interface. These interface effects alter the energy band alignment and charge transfer characteristics, as shown in Figure 5a, leading to changes in device performance and behavior changes. Thicker LiF layers can lead to stronger dipole formation at the LiF/MoS_2_ interface. This dipole can create an electric field that affects the behavior of the charge carriers in the channel. Changes in the electric field, induced by the presence of dipoles, can impact the mobility of charge carriers, consequently influencing the overall FET performance.^61^ Furthermore, dipoles near the channel can lead to shifts in the threshold voltage of the FET, representing the gate voltage, for which the FET transitions from the off to the on state. Such shifts may indicate alterations in charge carrier concentration or mobility influenced by the dipole presence. Understanding the interplay between dipoles and the electric field in the channel is imperative for optimizing the performance of FETs and other electronic devices. The application of the LiF layer as a dielectric has not been extensively explored, and the relevant literature is challenging to further investigate. Nevertheless, theoretical explanations were provided regarding dipole alignment between MoS_2_ and hBN, where the hBN was used as the dielectric. Depending on the direction and strength of the dipole, it can either enhance or hinder charge carrier transport. In some cases, it can lead to a less desirable transistor behavior, as observed in the case of a 20-nm-thick LiF layer encapsulated channel of a MoS_2_ FET. The optimized thickness of the LiF capping layer is necessary to achieve the desired device performance. Since the 10-nm-thick LiF layer as the capping layer proved extremely beneficial, we further studied its impact under different voltage biases of *V*_d_ = 0.1, 1, 2, 3, and 4 V, respectively. The mobility at the other voltage biases with and without capping layers was calculated from the transfer curves (Figure S9) and plotted in Figure 5b. Shift in the *V*_th_ was observed at the gate biasing shown in Figure 5c, as calculated from the transconductance curve (*g*_m_), as shown in Figure S10. The max on-current (*I*_on_) = 312 μA was obtained at *V*_d_ = 4 V, as depicted in Figure 5d and e. A decent decrease in SS can be observed at all *V*_d_ values (Figure 5f). We extracted the contact resistance (*R*_c_) for further investigation, because contact properties strongly affect electrical characteristics. Several methods are currently employed for evaluating contact resistance, including the transfer-line method (TLM), gated four-probe measurement, and Kelvin probe force microscopy (KFM).^17,62,63^ TLM is a widely used method that allows for the observation of contact resistance changes with the gate voltage. However, it requires multiple transistors with varying channel lengths, resulting in an average contact resistance value for the transistor set. It is not suitable for triangular-shaped MoS_2_ due to difficulties in creating uniform contacts with varying channel lengths, making it unsuitable for small flake sizes. The gated four-probe measurement also faces challenges with channel nonuniformity. On the other hand, KFM is excellent for analyzing contact resistance, particularly in distinguishing the contributions of the source and drain. However, it is more complex compared to conventional current–voltage (*I*–*V*) characterizations. To overcome these limitations, the Y-function method (YFM) was proposed for contact resistance extraction in CVD-grown monolayer MoS_2_-based FETs. The YFM was originally established for parameter extraction in silicon metal–oxide–semiconductor field-effect transistors (MOSFETs) and has shown accuracy and simplicity in low-field mobility and threshold voltage extraction.^64^ The extracted *R*_c_ shows reduction from 9 kΩ·μm to 5 kΩ·μm, as shown in Figure S11. This reduction in the *R*_c_ can greatly affect electron injection from the metal into the semiconductor. Without the LiF capping layer, the high barrier height initially hinders electron injection. After capping by the LiF layer, electron injection becomes easier, leading to an increase in current through the metal-to-semiconductor junction (Figure 5a). However, the value of contact resistance is still relatively higher than the reported values and cannot be compared fairly for several reasons. It is important to highlight that the computed *R*_c_ derived from the Y-function represents an upper limit, and the actual *R*_c_ may potentially be lower.^65−67^ Also, our treatment was confined to only the channel. However, there are still some fair chances of diffusion of the alkali fluoride particles around the contact edges.

To ensure that the heightened device performance is not solely contingent on the contact shape, we employed a patterning approach for our CVD-grown MoS_2_ triangles. The devices were fabricated with a channel length (*L*_ch_) of 10 μm and a channel width (*W*_ch_) of 6 μm, as illustrated in Figure S12a. Subsequently, Figure S12b shows the corresponding patterned device with LiF capping. To achieve this patterning of MoS_2_, the target substrate underwent lithography to generate the required pattern, following which the MoS_2_ sample was successfully transferred from the growth substrate to the target substrate, selectively adhering to the designated regions. To probe the impact of LiF doping on MoS_2_ FETs, electrical measurements were conducted before and after the application of the capping layers, as depicted in Figure S12c. The current density at *V*_d_ = 1 V exhibited a notable increase from approximately 5 μA/μm to about 21 μA/μm for the monolayer MoS_2_ back-gated FETs, underscoring the positive effect of the LiF layer capping. In addition, the carrier mobility (μ) was determined, revealing an increase from 25 cm^2^/(V s) (pristine MoS_2_) to 98 cm^2^/(V s) in the 10 nm LiF-capped MoS_2_ FETs at *V*_d_ = 1 V. Furthermore, to investigate the hysteric behavior in the transfer characteristics of the MoS_2_ FET, we employed a dual-sweep mode for gate voltage application. Initially, the device was exposed to ambient conditions without any capping to observe the inherent hysteretic nature under different gate bias stresses. The gate voltage was swept from a negative value of −10 V to a high positive value of 35 V and then swept back to −10 V in a cyclic manner, as illustrated in Figure S13a, resulting in a clockwise hysteresis. The width of the observed hysteresis (Δ*V*) was measured as the maximum threshold voltage shift in the transfer characteristics between backward and forward sweeps.^68^ This dual sweep was conducted at a constant drain voltage (*V*_d_) of 1 V to examine potential variations in the hysteresis width. A substantial width is consistent with previous reports, which attribute it primarily to a significant accumulation of charge trapping at the MoS_2_ surface due to the adsorption/desorption of ambient gases and water molecules.^23,69^ To mitigate the influence of these external factors, we proceeded with the hysteresis study with a LiF-capped MoS_2_ FET channel, maintaining the same gate bias sweeping conditions, as illustrated in Figure S13b. Comparative analysis of the hysteresis curves reveals a reduction in hysteresis width from Δ*V* = 2 V to Δ*V* = 0.5 V. The residual hysteresis is primarily attributed to oxide trapping at the MoS_2_/SiO_2_ interface, arising from unavoidable dangling bonds at the SiO_2_ surface.^70^ The population of trapped charges (*D*_it_) can be quantified using the equation , where *C*_ox_ represents oxide capacitance and *q* is the elementary charge^25,71^ The number of trapped charges decreased from 8.6 × 10^11^ cm^–2^ (pristine MoS_2_ channel) to 2.1 × 10^11^ cm^–2^ (LiF-passivated MoS_2_ channel.)

## Conclusion

In conclusion, we have used alkali metal fluoride dielectric capping layers, including LiF, NaF, and KF dielectric capping layers, to mitigate the environmental impact of oxygen and water exposure. Among them, the emergence of the LiF layer as a superior choice for a capping layer on MoS_2_ FETs offers excellent electrical insulation properties, acting as an effective dielectric layer for MoS_2_ devices and improving the overall performance of the FETs. The high bandgap of the LiF capping layer and low electron affinity make it an ideal material as the capping layer on the MoS_2_ channel, preventing charge carriers from escaping or interacting with the environment, thereby enhancing device stability and reliability. Additionally, the deposition of the LiF capping layer is relatively straightforward and compatible with existing fabrication processes. This compatibility with standard fabrication processes simplifies integration into existing manufacturing workflows, facilitating the large-scale production of MoS_2_ FETs. The LiF capping layer demonstrates promising potential to improve the electronic properties of MoS_2_ devices. It has been observed to induce n-type doping in the MoS_2_ channel, as confirmed by electrical measurements and surface XPS analysis, enhancing the overall device performance and enabling the design of more efficient and versatile circuits. The mobility significantly increased from 74 cm^2^/(V s) to 137 cm^2^/(V s), and the current density increased significantly from 17 μA/μm to 32.13 μA/μm at a *V*_d_ of 1 V with the subthreshold swing decreasing to 0.8 V/dec. This doping effect provides possibilities for MoS_2_-based electronics and highlights the advantages of the LiF layer as a capping material. These findings highlight certain important trade-offs and provide insight into the significance of interface control and passivation material choice on the electrical stability, performance, and enhancement of MoS_2_ FETs.

## Methods

### Synthesis of MoS_2_

Molybdenum(VI) oxide (MoO_3_) powder (Alfa Aesar; 99.95%) and sulfur powder (Aldrich; 99.5–100.5%) served as the source materials for the CVD growth of MoS_2_ on sapphire substrates (Figure S1). To ensure the successful deposition, substrates were ultrasonicated in a sequence of solvents: acetone, isopropanol, and, finally, deionized water, each for a duration of 10 min, respectively, prior to deposition. Sapphire substrates and 2 mg of MoO_3_ powder were placed in separate holders in the middle of the quartz tube, while the sulfur powder was placed upstream. A 1 mg amount of sodium chloride (NaCl; Showa; 99.5%) was mixed with MoO_3_ powder as a catalyst for MoS_2_ growth. Initially, the quartz tube was evacuated to a base pressure of 200 mTorr, creating a low-pressure environment. Then, ultrahigh-purity argon gas (Ar) was introduced at a flow rate of 30 sccm, and the working pressure was raised to 560 Torr. The furnace was then heated to a temperature of about 850 °C, while sulfur was separately heated to 180 °C using an external heating belt. The elevated temperatures facilitate the chemical reactions necessary for the MoS_2_ synthesis. After the growth period of 10 min, the CVD furnace was gradually cooled down to a temperature of 100 °C, and at this point, the samples were carefully extracted from the furnace. This controlled process facilitated the successful deposition of MoS_2_ on the sapphire substrate.

### Transfer Method

The transfer of the 2D material was achieved through a three-step method involving separation, fishing up, and removal (Figure S2). First, PMMA (Kayaku, 950 PMMA A4) was coated on the as-grown 2D material using a spin coater at 800 rpm for 10 s, followed by an increased speed of 2000 rpm for 30 s to serve as a supporting layer. The higher speed at the end helps to ensure a uniform and smooth layer. The edges of the film were then gently scraped with tweezers to minimize any damage to the film and make a space between the grown substrate and PMMA/2D stack. This gap was important for the subsequent separation process. A dilute ammonia solution (NH_4_OH/DI water = 1:5) was used to separate the edges. In the final section of fishing up, the film was carefully detached and subsequently cleaned with DI water. The floating delaminated stack of PMMA/2D material was then fished up by using the clean target substrate. In the final section of removal, the water droplet caught between the substrate and film was removed by heating it for 20 min at 70 °C. This technique guarantees an improved 2D material adherence to the desired substrate. Postbaking, the entire substrate was immersed in acetone solution for 30 min at room temperature to dissolve the PMMA layer. Following the dissolution of PMMA, any remaining residue and contaminants were removed using isopropyl alcohol (IPA) and DI water.

### MoS_2_ Field-Effect Transistor Device Fabrication

The successfully transferred MoS_2_ triangles and thin film on p+Si/SiO_2_(50 nm) were further utilized for the back-gate FET fabrication. Direct light patterning (DLP) was used to define the channel and source/drain with S18134 photoresist. To better compare the electrical performance of devices, we preferred to maintain the same channel length of 10 μm. After the exposure, the pattern was developed using the AD-10 developer. Metallization is carried out by electron beam evaporation at a deposition rate of 0.3A s^–1^ at ∼10^–6^ Torr. The final step is the lift-off process, during which the device was immersed in a PG remover to remove the photoresist, followed by rinsing with acetone, then IPA, and finally DI water. To ensure the deposition of the alkali metal at the specified channel regions, a second lithography was conducted to pattern the area. The LiF/AL_2_O_3_ was deposited using e-beam evaporation.

### Measurements and Characterization

The topography of the MoS_2_ sample was first characterized by using optical microscopy (OM). Raman and PL spectroscopy were performed using a HORIBA LabRAM HR800 using a 532 nm laser at a power of 50 mW, while the Si peak at 520 cm^–1^ served as the standard reference peak. To study the charge transfer between Li and MoS_2_, XPS surface analysis was performed. HRTEM (JEOL, JEM-F200, 200 kV) at 200 keV was used to obtain cross-sectional images of transferred and doped MoS_2_. Device electrical measurements were carried out at room temperature using a semiconductor parameter analyzer (Agilent, B1500A).

## Supporting Information Available

Summary of electronegativity, electron affinity and ionization energy values for Li, Na, K, and F in the LiF, NaF, and KF compounds; schematics for the CVD synthesis of monolayer MoS_2_ flakes; schematics for the wet transfer of the CVD-grown MoS_2_; XPS spectrum after the capping of the NaF layer; performance characterization of the MoS_2_ transistors before and after the capping of the 10-nm-thick NaF layer on MoS_2_ FETs; performance characterization of the MoS_2_ transistors before and after the capping of the 10-nm-thick KF layer on MoS_2_ FETs; performance characterization of the MoS_2_ transistors with the capping of a 20-nm-thick Al_2_O_3_ layer; *I*_d_–*V*_g_ characteristics of MoS_2_ FETs with different thicknesses; log scale transfer characteristics and threshold voltages of MoS_2_ FETs before and after the capping of LiF layers with different thicknesses; *I*_d_–*V*_g_ characteristics of the MoS_2_ before and after capping a 10-nm-thick LiF layer at different *V*_d_; transconductance curves before and after the capping of the 10-nm-thick LiF layer at different *V*_d_; Y function before and after the capping of the 10-nm-thick LiF layer (PDF)
