# Supplementary material for: Enhanced Electrical Transport Properties of Molybdenum Disulfide Field-Effect Transistors by Using Alkali Metal Fluorides as Dielectric Capping Layers
Source: ACS Nano. 2024 Apr 8;18(16):10776–87. doi: 10.1021/acsnano.3c11025 (PMC11044573; doi:10.1021/acsnano.3c11025)
Supplement: Supplementary file 1 — nn3c11025_si_001.pdf [file nn3c11025_si_001.pdf]

Supplementary Information

Enhanced Electrical Transport Properties of Molybdenum Disulfide Field-Effect Transistors by Using Alkali Metal Fluorides as Dielectric Capping Layers

Sumayah-Shakil Wani[1, 3, 5], Chen Chieh Hsu[2], Yao-Zen Kuo[1, 3, 5], Kimbulapitiya Mudiyanselage Madhusanka Darshana Kumara Kimbulapitiya[1, 3, 5], Chia-Chen Chung[1, 3, 5], Ruei-Hong Cyu[1, 3, 5], Chieh-Ting Chen[1, 3, 5], Ming-Jin Liu[1, 3, 5], Mayur Chaudhary[1, 3, 5], Po-Wen Chiu[3, 4], Yuan-Liang Zhong[2*], Yu-Lun Chueh[1, 3, 5, 6*]

[1]Department of Materials Science and Engineering, National Tsing-Hua University, Hsinchu 30013, Taiwan.

[2]Department of Physics and Quantum Information Center, Chung Yuan Christian University, Taoyuan, 32034, Taiwan

[3]College of Semiconductor Research, National Tsing-Hua University, Hsinchu, 30013, Taiwan.

[4]Institute of Electronics Engineering, National Tsing Hua University, Hsinchu 30013, Taiwan

[5]Department of Physics, National Sun Yat-Sen University, Kaohsiung, 80424, Taiwan.

[6]Department of Materials Science and Engineering, Korea University, Seoul 02841, Republic of Korea.

*E-mail: ylchueh@mx.nthu.edu.tw and ylzhong@cycu.edu.tw

Table 1: Summary of electronegativity, electron affinity, and ionization energy values for Li, Na, K, and F in the LiF, NaF, and KF compounds by unit eV.[1]

Properties	Li	Na	K	F
Electronegativity	0.98	0.93	0.82	3.98
Electron affinity	0.62	0.55	0.50	3.40
Ionization Energy	5.39	5.14	4.34	17.42

(1) B. D. Pelatt, R. Ravichandran, J. F. Wager, D. A. Keszler, *Journal of the American Chemical Society* 2011, 133, 16852.

(2) L. C. Allen, *Journal of the American Chemical Society* 1989, 111, 9003.

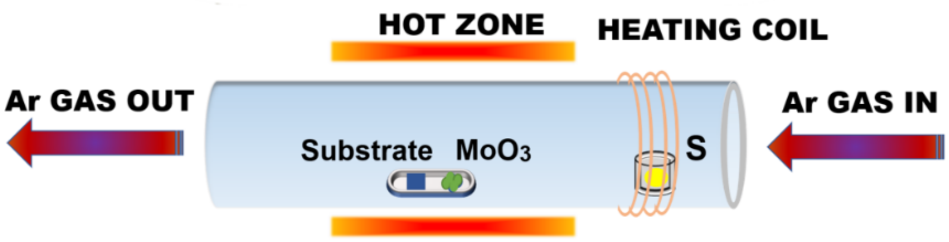

Figure S1. Schematics for the CVD synthesis of monolayer MoS_2 flakes.

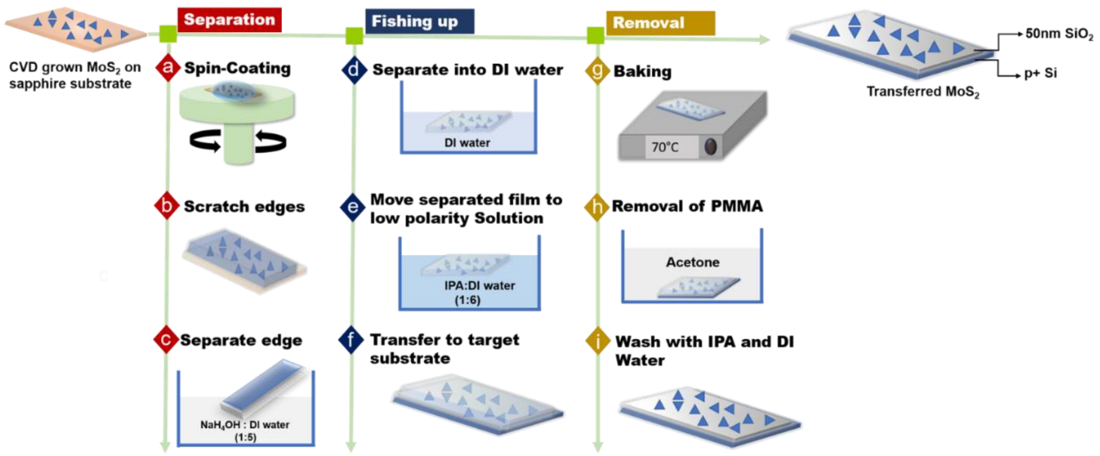

Figure S2. Schematics for the wet transfer of the CVD-grown MoS_2.

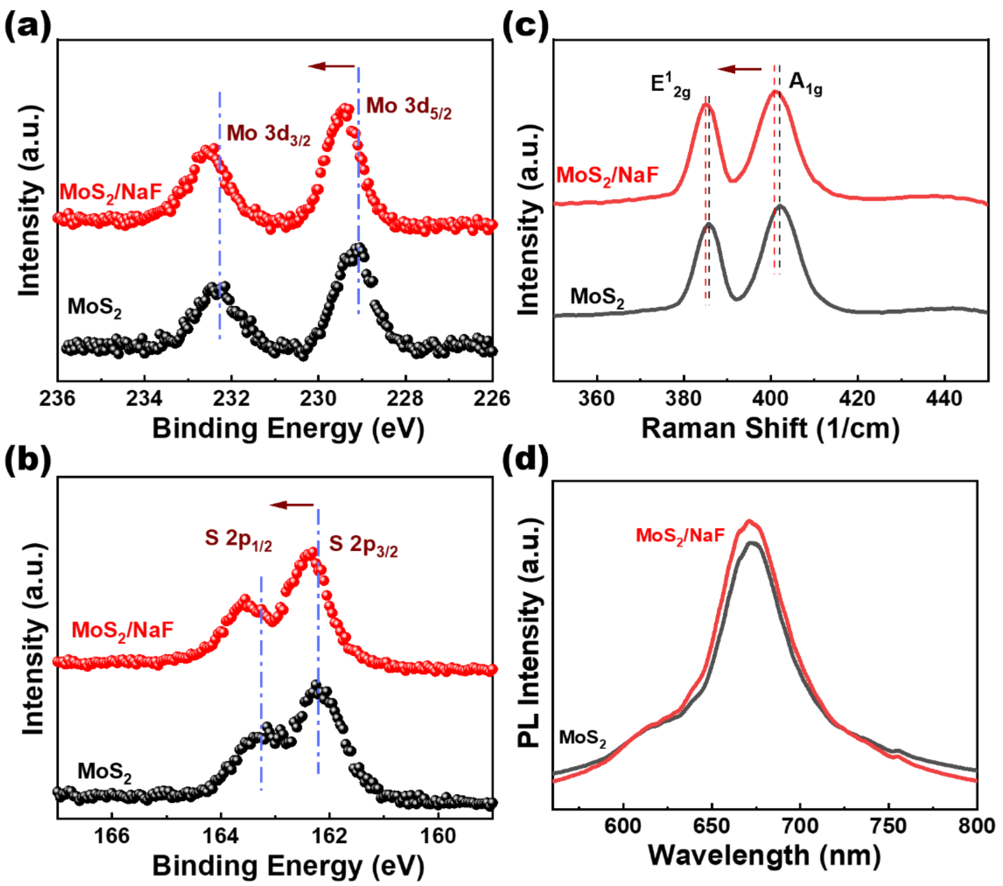

Figure S3. X-ray photoelectron spectroscopy (XPS) spectrum of (a) Mo 3d and (b) S 2p before and after the capping of the NaF layer. The shift of the binding energies to higher energy indicates n-type doping. (c) Raman spectra of MoS_2 before and after the capping of the NaF layer. The capping of the NaF layer induces a red-shift behavior on (d) photoluminescence (PL) measurements of MoS_2 before and after the capping of the NaF layer.

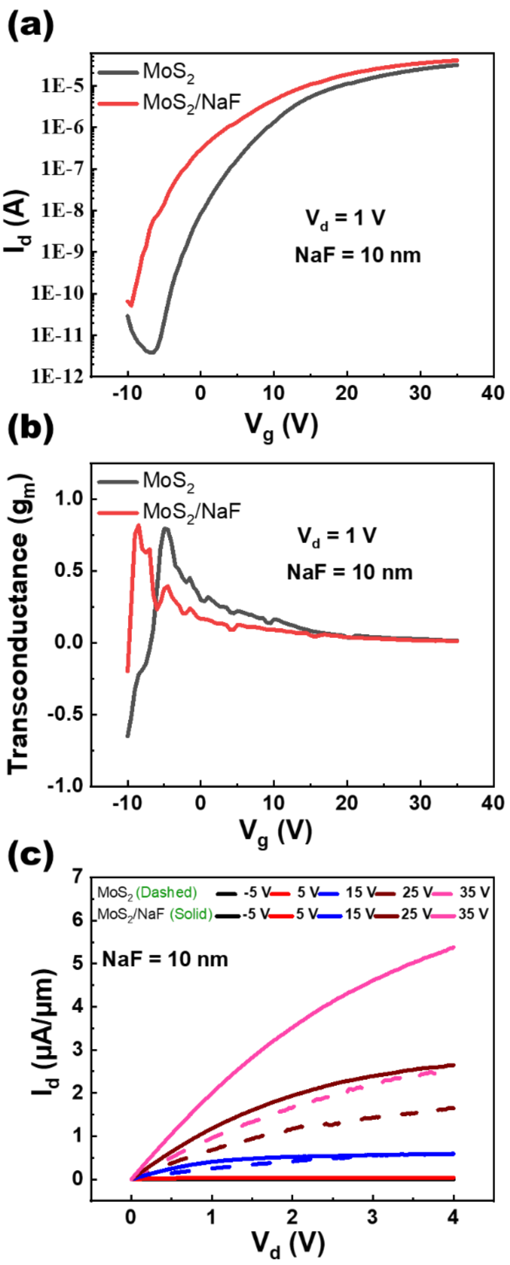

Figure S4. Performance characterization of the MoS_2 transistors before and after the capping of the 10 nm-thick NaF layer on MoS_2 FETs: (a) Log scale I_d–V_g characteristics, (b) transconductance plots, and (c) output characteristics I_d-V_d of MoS_2 FETs with and the capping of the 10 nm-thick NaF layer.

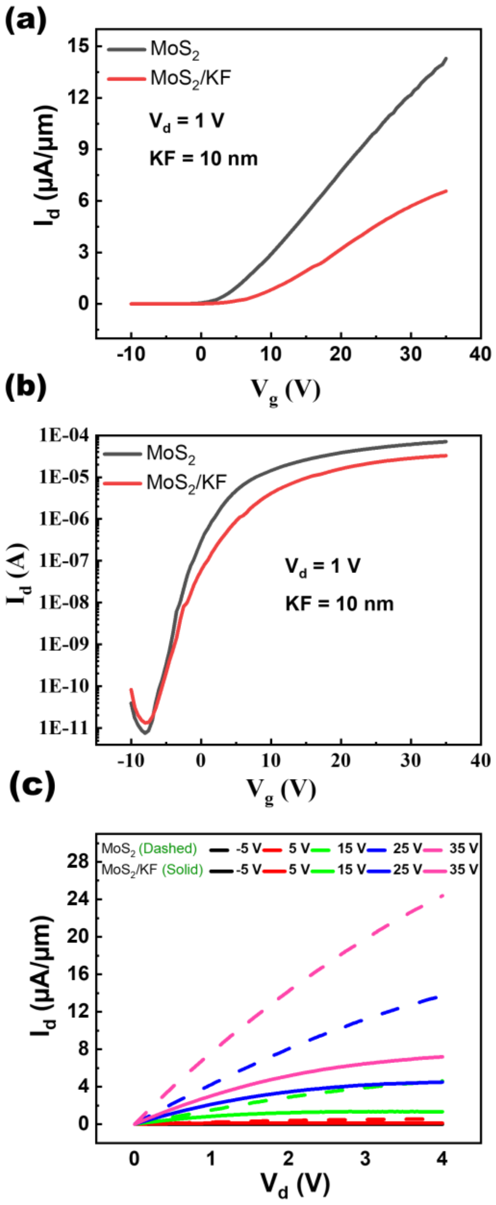

Figure S5. Performance characterization of the MoS_2 transistors before and after the capping of the 10nm-thick KF layer on MoS_2 FETs: (a) I_d–V_g Transfer characteristics, (b) logscale I_d-V_g plots, and (c) I_d-V_d characteristics of MoS_2 FETs with and without the capping of the 10nm-thick KF layer.

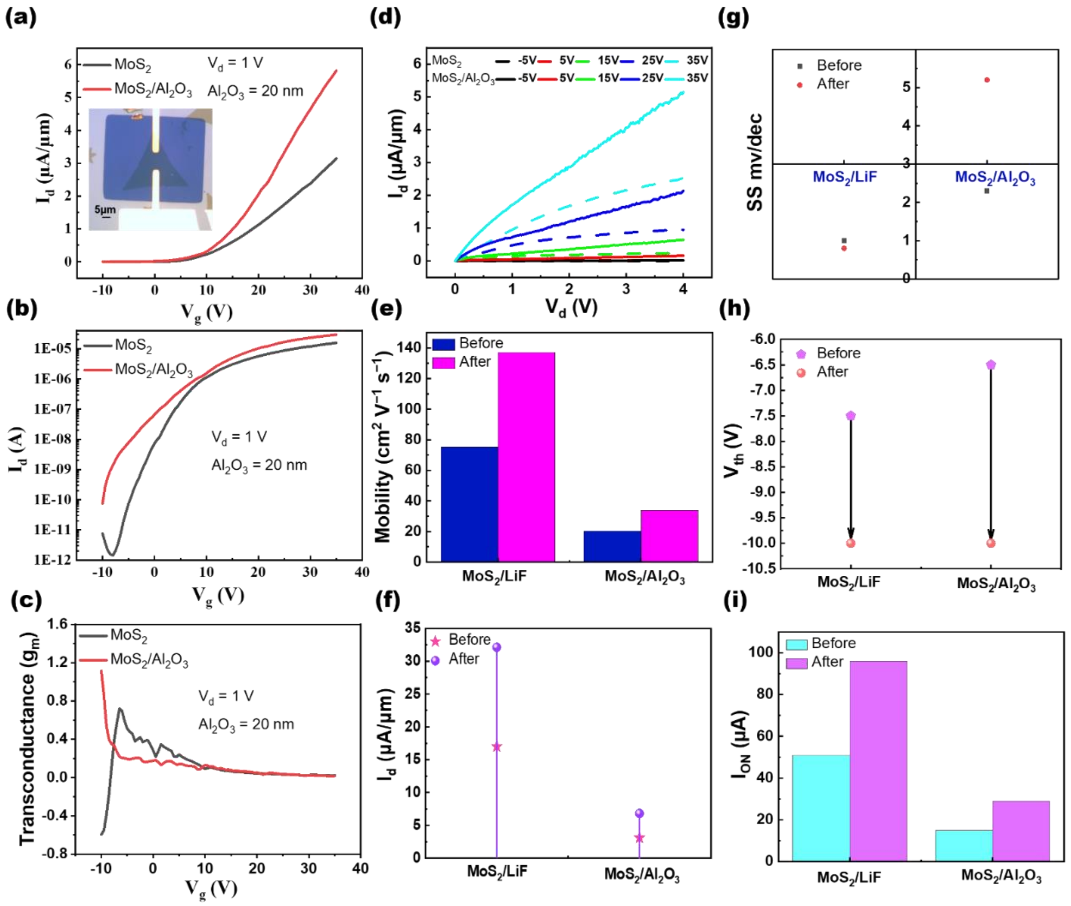

Figure S6. Performance characterization of the MoS_2 transistors with the capping of 20 nm-thick Al_2O_3 layer: (a) I_d–V_g Transfer characteristics. Inset shows the corresponding OM image. (b) logscale I_d–V_g plots, (c) transconductance plots, and (d) output characteristics (dashed and solid line corresponds to MoS_2 without and with Al_2O_3 capping). Electrical performance comparison on (e) mobility measurements, (f) charge carrier density, (g) subthreshold swing, (h) threshold voltage shift, and (f) on-current with the capping of the LiF and Al_2O_3 layers on MoS_2, respectively.

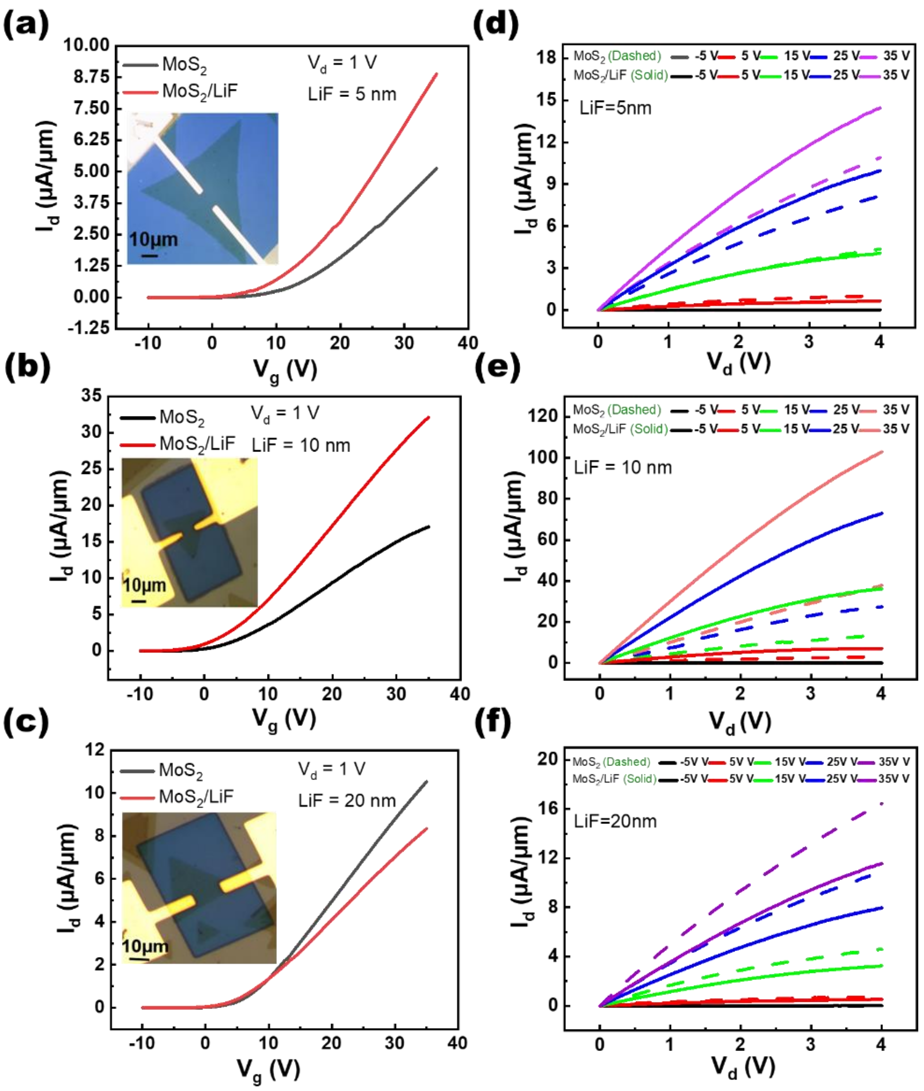

Figure S7. I_d–V_g characteristics of MoS_2 FETs with different thicknesses of (a) 5 nm-thick, (b) 10 nm-thick, and (c) 20 nm-thick LiF capping layers. I_d-V_d characteristics of the MoS_2 FET with (d) 5 nm-thick, (e) 10 nm-thick, and (f) 20 nm-thick LiF capping layers.

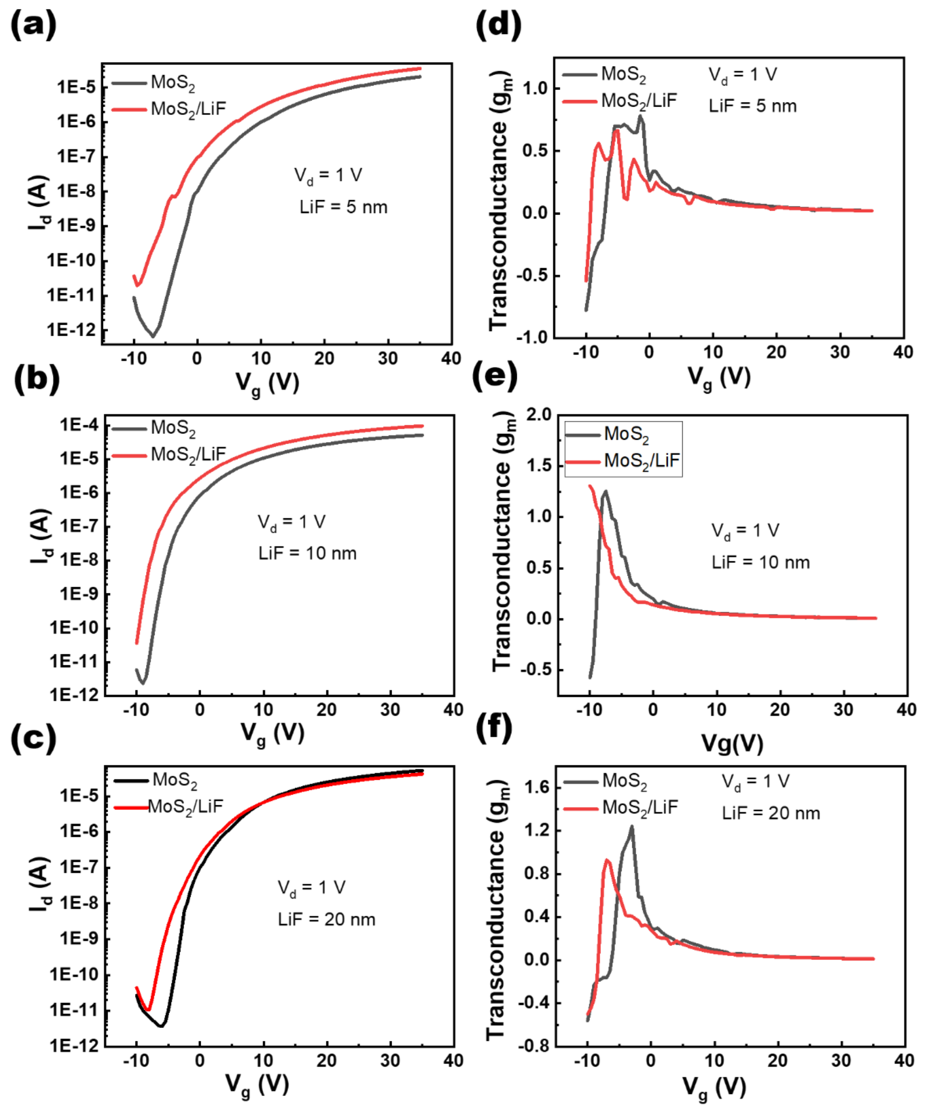

Figure S8. Log scale transfer characteristics and threshold voltages of MoS_2 FETs before and after the capping of (a) 5 nm-thick, (b) 10 nm-thick, and 20 nm-thick LiF layers, respectively.

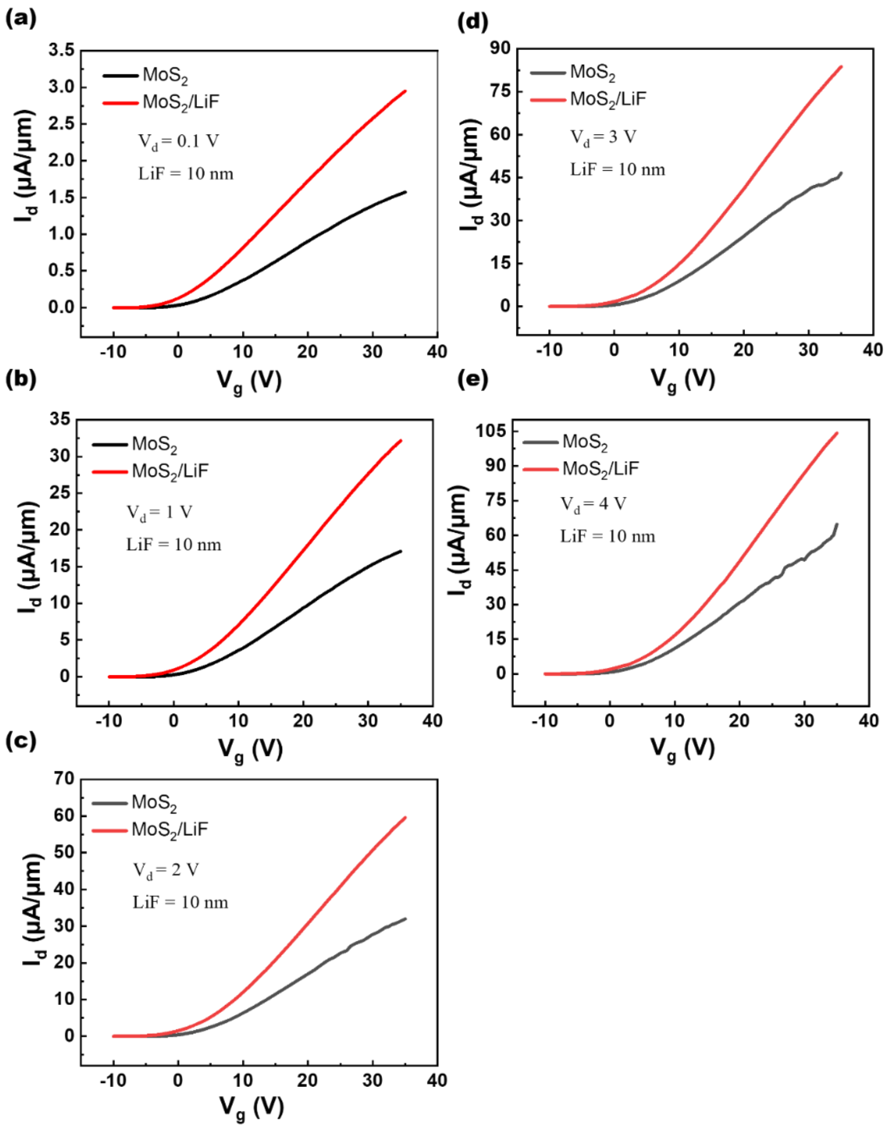

Figure S9. I_d–V_g characteristics of the MoS_2 before and after 10 nm-thick LiF layer at different V_d of (a) 0.1, (b) 1, (c) 2, (d) 3, and (e) 4 V, respectively.

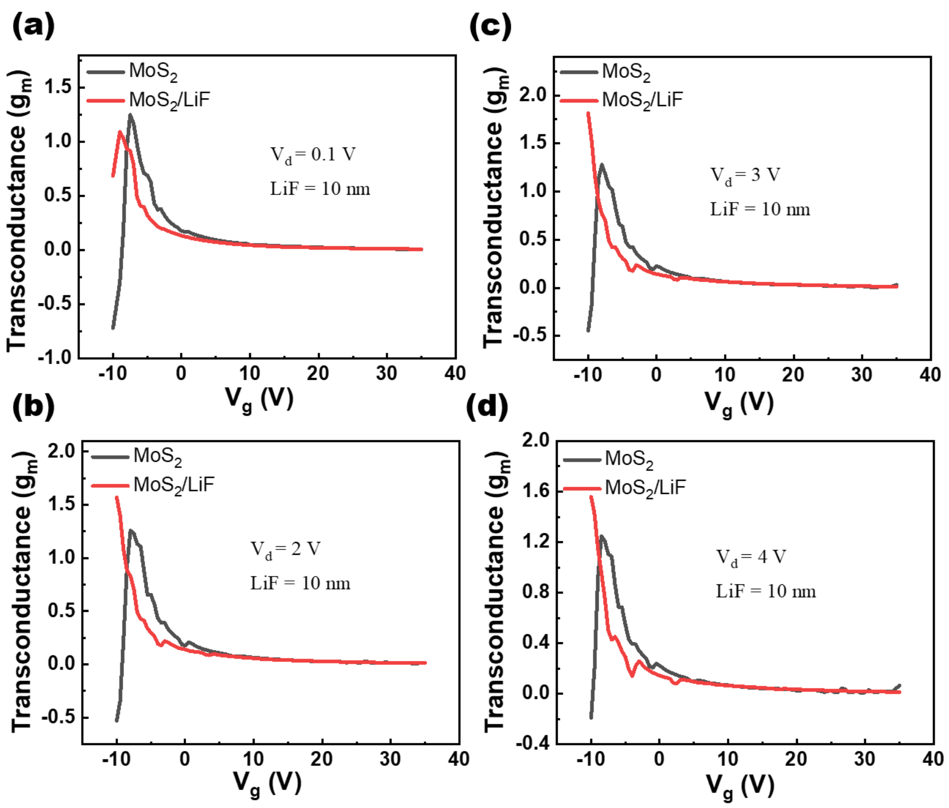

Figure S10. Transconductance curves before and after the capping of the10 nm-thick LiF layer at V_d of (a) 0.1, (b) 2, (c) 3, and (d) 4 V, respectively.

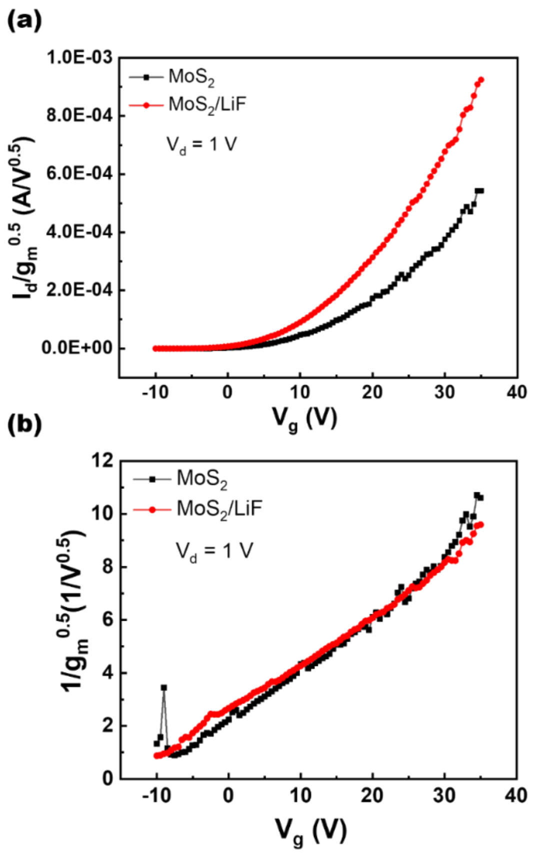

Figure S11. Y function before and after the capping of the 10 nm-thick LiF layer. (a) Plots of Y function with respect to V_{gs} and the linear fitting in the strong accumulation region for obtaining the slope. (b) Plots of 1/gm 0.5 with respect to the V_{gs}.

Supplementary note:

Y-function model

The Y-function method relies on analyzing the drain current (I_d) in the linear region by applying a large gate voltage and a small source-drain voltage ($V_d << V_g$). The Y-function method is a straightforward approach that analyzes the drain current (I_d) in the linear region by applying a large gate voltage and a small source-drain voltage $V_d << V_g$ [2]. The linear region drain current (I_d) can generally be described by the equation:

$$I_d = \frac{\mu_0}{1+\theta(V_g - V_{th})} C_i \frac{W}{L}(V_g - V_{th})V_d \quad (1)$$

Where μ_0, C_i, V_{th}, W, L, and θ represent the effective mobility in the linear regime, the intrinsic mobility, the capacitance between the channel and the gate per unit area, the threshold voltage, the channel width, the channel length, and the mobility attenuation coefficient, respectively. The Y-function, denoted by Y, is defined based on the transconductance ($g_m = \partial I_d/\partial V_g$) and can be expressed as:

$$Y = \frac{I_d}{\sqrt{g_m}} = \frac{I_d}{\sqrt{I_d/[1+\theta(V_g - V_{th})](V_g - V_{th})}} = \sqrt{\mu_0 C_i V_d \frac{W}{L}}(V_g - V_{th}) \quad (2)$$

The value s_1 is extracted from the slope of the Y-function versus V_g, while s_2 is obtained from the slope of $1/\sqrt{g_m}$ versus V_g. The contact resistance (R_c) can be calculated using the equation:

$$R_c = \frac{s_2}{s_1} V_d \quad (3)$$

References:

(1) Pelatt, B. D.; Ravichandran, R.; Wager, J. F.; Keszler, D. A. Atomic solid state energy scale. *Journal of the American Chemical Society* **2011**, *133* (42), 16852-16860. Allen, L. C. Electronegativity is the average one-electron energy of the valence-shell electrons in ground-state free atoms. *Journal of the American Chemical Society* **1989**, *111* (25), 9003-9014.

(2) Chang, H.-Y.; Zhu, W.; Akinwande, D. On the mobility and contact resistance evaluation for transistors based on MoS2 or two-dimensional semiconducting atomic crystals. *Applied Physics Letters* **2014**, *104* (11), 113504.

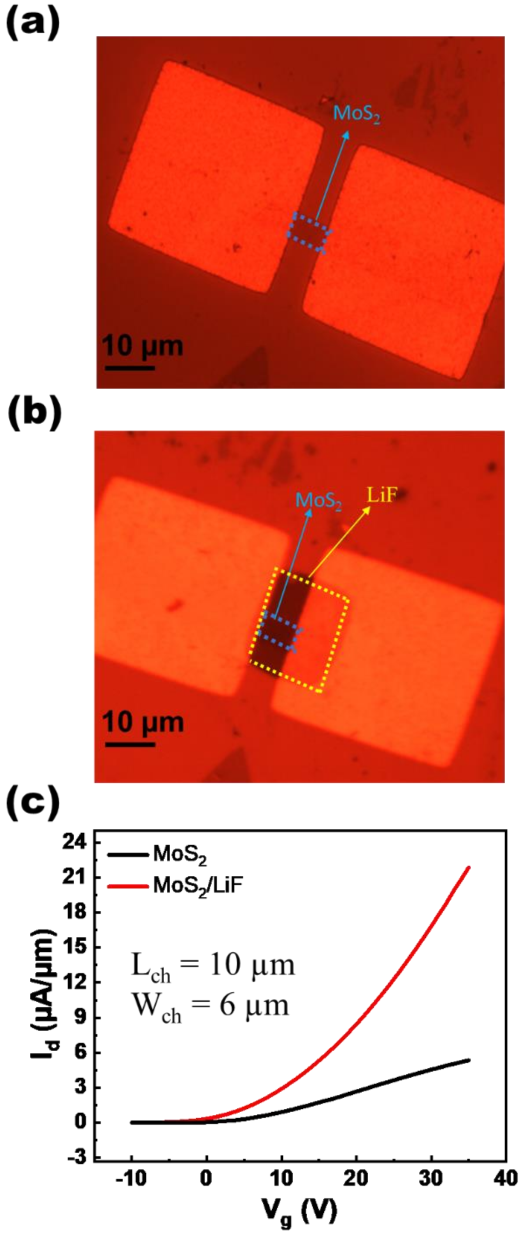

Figure S12. An optical microscopic image shows a fabricated monolayer MoS_2 FET (a) without capping layer (b) with 10nm LiF passivation layer. (c) I_d–V_g Transfer characteristics V_{ds} =1 V of patterned MoS_2 transistors before and after the capping of the 10 nm-thick LiF layer.

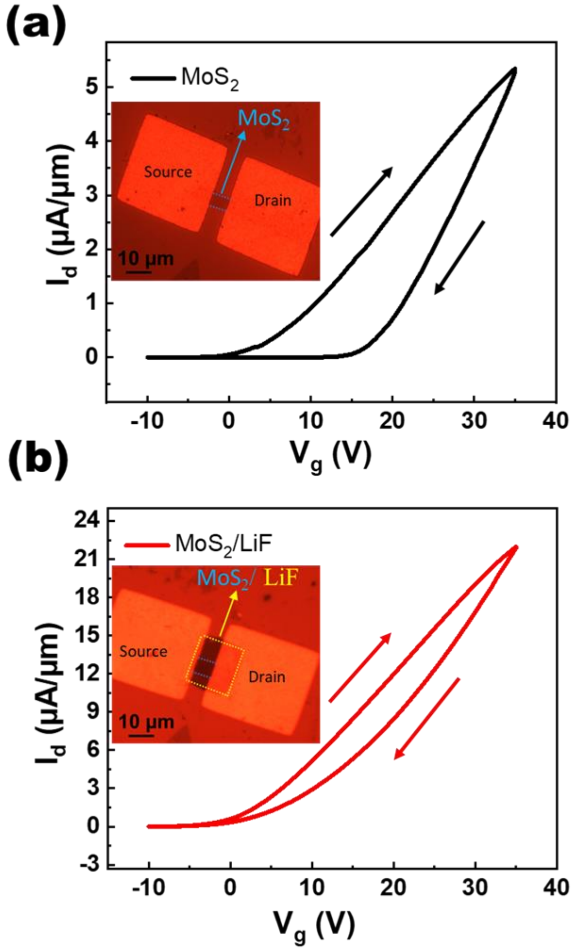

Figure S13. Hysteresis loops in the transfer characteristics of MoS_2 FET for gate voltage sweep range -10 V to 35 V (a) Pristine MoS_2 FET and (b) MoS_2 channel passivated by LiF/Al_2O_3. (Since Al_2O_3 is only the protection layer for LiF, we have not mentioned it in the figure legend).
